# Changes in Hospital-Based Care Seeking for Acute Mental Health Concerns Among Children and Adolescents During the COVID-19 Pandemic in Ontario, Canada, Through September 2021

**DOI:** 10.1001/jamanetworkopen.2022.20553

**Published:** 2022-07-07

**Authors:** Natasha Ruth Saunders, Therese A. Stukel, Rachel Strauss, Longdi Fu, Eyal Cohen, Astrid Guttmann, Alene Toulany

**Affiliations:** 1 The Hospital for Sick Children, Toronto, Ontario, Canada; 2 Department of Pediatrics, University of Toronto, Toronto, Ontario, Canada; 3 ICES, Toronto, Ontario, Canada; 4 Child Health Evaluative Sciences, SickKids Research Institute, Toronto, Ontario, Canada; 5 Institute of Health Policy, Management and Evaluation, The University of Toronto, Toronto, Ontario, Canada; 6 Temerty Faculty of Medicine, University of Toronto, Toronto, Ontario, Canada; 7 Edwin S. H. Leong Centre for Healthy Children, University of Toronto, Toronto, Ontario, Canada

## Abstract

This cross-sectional study assesses hospitalization and emergency department visit rates among children and adolescents seeking mental health care in pediatric and nonpediatric hospitals in Ontario, Canada, before and during the COVID-19 pandemic.

## Introduction

The extent of the mental health effects of the COVID-19 pandemic on children and adolescents is controversial. Freestanding pediatric hospitals and others have reported increasing rates of mental illness following the pandemic’s onset^1,2,3^ that are not reflected in population data.^4,5,6^ Accurate data reports are needed to adequately support pediatric mental health care needs. We measured the changes in emergency department (ED) visits and hospitalizations for mental health diagnoses among children and adolescents in pediatric and nonpediatric hospitals before and during the pandemic.

## Methods

This population-based, repeated cross-sectional study of acute care visits among all Ontarians ages 3 to 17 years (approximately 2.5 million individuals) was conducted from January 1, 2017, to September 30, 2021, in Ontario, Canada. ED visits (through September 30, 2021) and hospital discharges (through August 28, 2021) with mental health diagnoses were identified using hospital and ED discharge records (eAppendix in the Supplement). The Registered Persons Databases was used to determine the annual population denominator. Use of these data was authorized under Ontario’s privacy legislation and was therefore exempt from institutional review board approval. This study followed the Reporting of Studies Conducted Using Observational Routinely Collected Health Data (RECORD) reporting guideline.

Weekly mental health–related ED visit and hospitalization rates per 10 000 population for pediatric hospitals, which are freestanding tertiary centers, and nonpediatric hospitals were calculated in the 3 years before and 18 months after pandemic onset. Poisson generalized estimating equations were used to model the 3-year prepandemic trends and estimate postpandemic onset trends. Visit rate changes following the pandemic onset were estimated as with our previous work.^4^ Statistical analyses were conducted using SAS, version 9.4 (SAS Institute Inc).

## Results

Based on the 3-year prepandemic baseline of almost 2.5 million children annually, expected weekly mental health-related ED visit rates were 0.39 per 10 000 (pediatric hospitals) and 1.93 per 10 000 population (nonpediatric hospitals). The expected weekly hospitalization rates were 0.18 per 10 000 (pediatric hospitals) and 0.55 per 10 000 population (nonpediatric hospitals).

**Figure 1.**
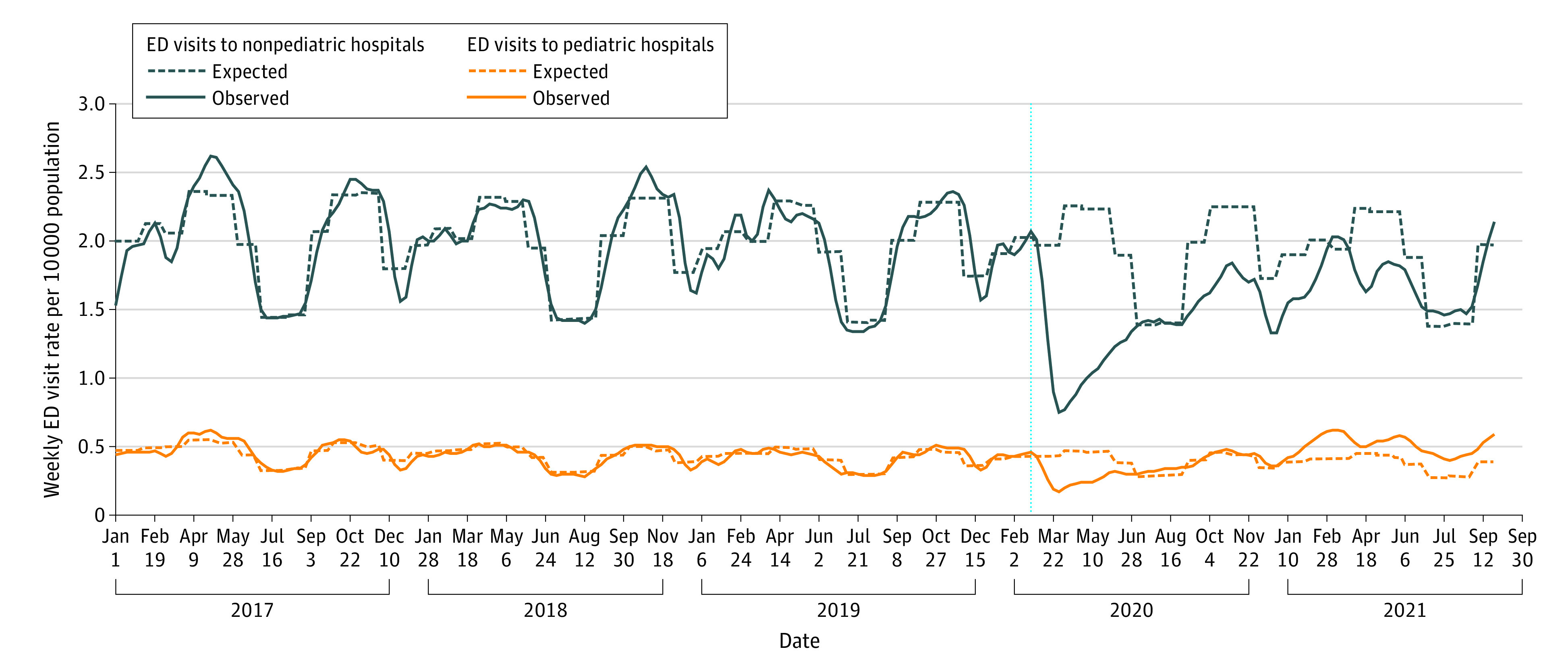
Pediatric Mental Health Emergency Department (ED) Visits by Pediatric and Nonpediatric Hospitals in Ontario, Canada

**Figure 2.**
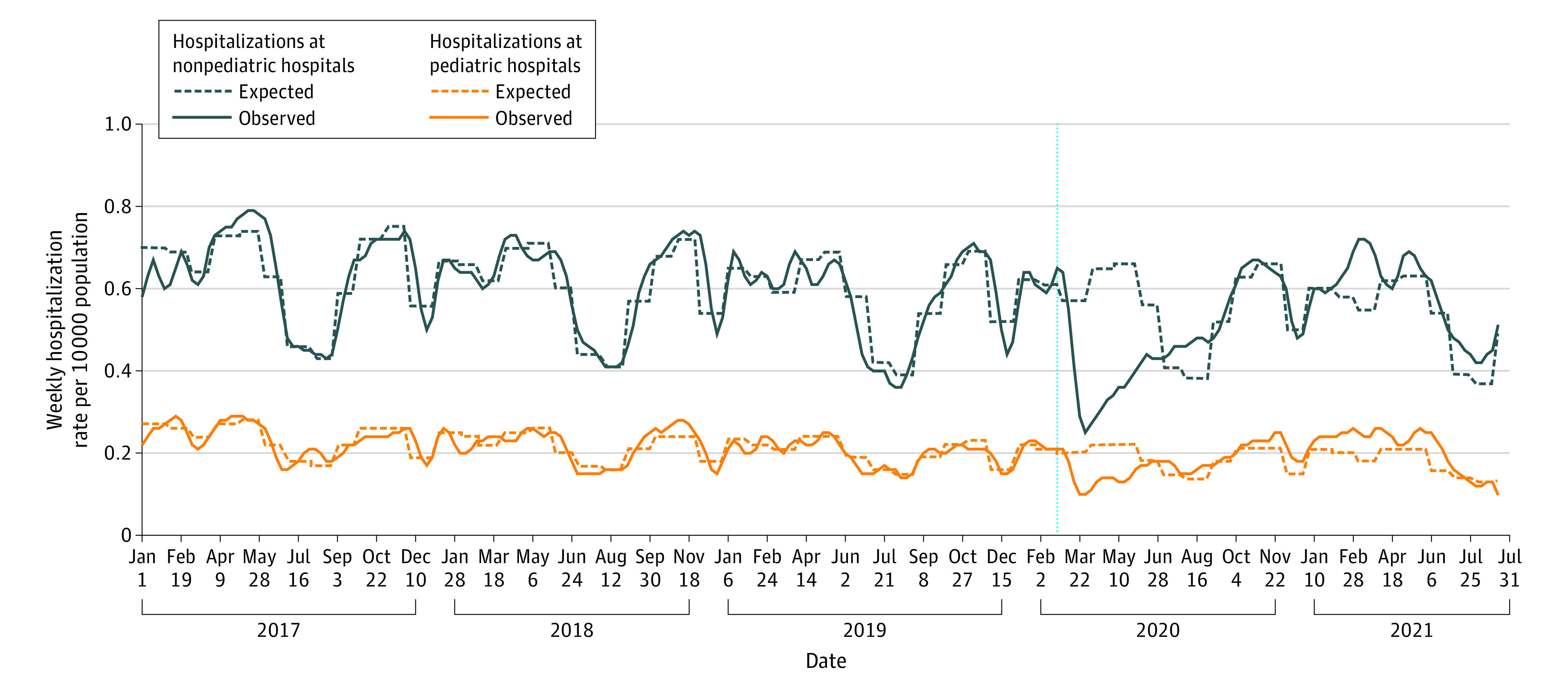
Pediatric Mental Health Hospitalizations by Pediatric and Nonpediatric Hospitals in Ontario, Canada

In the first 18 months of the pandemic, overall ED visit and hospitalization rates to pediatric hospitals were at expected levels compared with prepandemic (ED visits: adjusted relative rate (aRR), 1.08 [95% CI, 0.93-1.26]; hospitalizations: aRR, 1.05 [95% CI, 0.98-1.13]), although ED visits were consistently 41% to 62% above expected from February 2021 through the end of the study and hospitalizations were above expected from July 2020 to July 2021 (Figure 1 and Figure 2). In contrast, ED visit rates and hospitalizations at nonpediatric hospitals were 21% below and at expected levels, respectively (ED visits: aRR, 0.79 [95% CI, 0.75-0.84]; hospitalizations: aRR, 0.96 [95% CI, 0.91-1.01]). ED visits to nonpediatric hospitals remained below expected levels for most pandemic months, and hospitalizations remained at or below expected levels except for peaks in March, July, and August 2021.

## Discussion

In this population-based study in Ontario, Canada, we observed important differences in where children and adolescents received acute mental health care during the pandemic. For most of the pandemic, pediatric hospitals experienced higher than expected mental health visit rates, whereas nonpediatric hospitals experienced lower than expected rates, particularly in ED settings. It is unclear whether these patterns are due to changing demographics (eg, younger age) and/or clinical characteristics (eg, mental health diagnoses and comorbidities) among those seeking care, contextual dynamics specific to the Canadian health and social systems, or other factors.

Study limitations include our inability to measure unmet needs and drivers of care seeking. These data highlight the importance of context in understanding the pandemic-related changes to patterns of mental health care hospital use for children and adolescents. Understanding care-seeking behavior and the reasons why a shift toward accessing acute mental health care in pediatric hospitals occurred is essential to ensure that services match the needs of the populations served. Further, ongoing surveillance of patterns of acute mental health care delivery and system level planning are important to facilitate effective and efficient use and deployment of mental health resources.

## Notes

Supplement.
eAppendix.
