# Supplementary material for: Changes in Hospital-Based Care Seeking for Acute Mental Health Concerns Among Children and Adolescents During the COVID-19 Pandemic in Ontario, Canada, Through September 2021
Source: JAMA Netw Open. 2022 Jul 7;5(7):e2220553. doi: 10.1001/jamanetworkopen.2022.20553 (PMC9264033; doi:10.1001/jamanetworkopen.2022.20553)
Supplement: Supplement. — eAppendix. [file jamanetwopen-e2220553-s001.pdf]

Supplemental Online Content

Saunders NR, Stukel TA, Strauss R, et al. Changes in hospital-based care seeking for acute mental health concerns among children and adolescents during the COVID-19 pandemic in Ontario, Canada, through September 2021. *JAMA Netw Open*. 2022;5(7):e2220553. doi:10.1001/jamanetworkopen.2022.20553

eAppendix.

This supplemental material has been provided by the authors to give readers additional information about their work.

© 2022 Saunders NR et al. *JAMA Network Open.*

eAppendix

<u>Emergency department</u> codes are from the National Ambulatory Care Reporting System (NACRS). NACRS includes data for all hospital-based day surgeries and emergency departments in Ontario, Canada. Client visit data is collected at the time of service in each facility. Diagnostic codes reflect the "ED Discharge Diagnosis" code from the health record. Diagnoses reflect the "primary diagnosis", with the exception of intentional self-harm which may be in a secondary position and the nature of the injury (e.g., laceration) in the primary diagnostic position. Discharge diagnoses in NACRS use the International Classification of Diseases, 10th edition with Canadian enhancements diagnostic codes.

<u>Hospitalization</u> codes are from the Canadian Institutes for Health Information Discharge Abstract Database (CIHI-DAD) and the Ontario Mental Health Reporting System (OMHRS). These datasets capture administrative, clinical and demographic information on hospital discharges at all facilities in Ontario, Canada. CIHI-DAD diagnostic codes use the International Classification of Diseases, 10th edition with Canadian enhancements. Diagnostic codes used reflect discharge diagnoses most responsible for the hospital stay. The exception to this was when an intentional self-injury code was in a secondary diagnostic code position with the "nature of injury" in the primary/main diagnostic position. OHMRS hospital records reflect mental-health specific beds in mental health facilities in the Province of Ontario. Discharge diagnoses use the International Classification of Diseases, 9th edition with Canadian enhancements codes.

**Emergency department and hospital discharge diagnosis codes**		
**Category**	**ICD-9-CM code (OMHRS)**	**ICD-10-CA (DAD/NACRS)**
Any mental health and addictions in discharge record.	Any OMHRS discharge record except where discharge diagnosis includes 290.x (dementia) and 294.x (dementia) or where primary diagnosis is missing.	Primary (most responsible) diagnosis= F06-F99 (mental health codes)<br><br>or<br><br>Secondary diagnosis = X60-X84, Y10-Y19, Y28 (intentional self-injury codes) when primary diagnosis ≠ F06-F99

ICD-10-CA, International Classification of Diseases, 10th edition with Canadian enhancements; DSM-IV, Diagnostic and Statistical Manual, 4th revision; NACRS, National Ambulatory Care Reporting System; CIHI-DAD, Canadian Institute for Health Information's Discharge Abstract Database; OMHRS, Ontario Mental Health Reporting System.

© 2022 Saunders NR et al. *JAMA Network Open.*
